# Photocatalytic Generation of Singlet Oxygen by Graphitic Carbon Nitride for Antibacterial Applications

**DOI:** 10.3390/ma17153787

**Published:** 2024-08-01

**Authors:** Davida Briana DuBois, Isabelle Rivera, Qiming Liu, Bingzhe Yu, Kevin Singewald, Glenn L. Millhauser, Chad Saltikov, Shaowei Chen

**Affiliations:** 1 Department of Chemistry and Biochemistry, University of California, 1156 High Street, Santa Cruz, CA 95064, USA; dbsimpso@ucsc.edu (D.B.D.); ijrivera@ucsc.edu (I.R.); qliu40@ucsc.edu (Q.L.); byu46@ucsc.edu (B.Y.); ksingewa@ucsc.edu (K.S.); glennm@ucsc.edu (G.L.M.); 2 Department of Microbiology and Environmental Toxicology, University of California, 1156 High Street, Santa Cruz, CA 95064, USA; saltikov@ucsc.edu

**Keywords:** graphitic carbon nitride, photocatalysis, reactive oxygen species, singlet oxygen, antibacterial agent

## Abstract

Carbon-based functional nanocomposites have emerged as potent antimicrobial agents and can be exploited as a viable option to overcome antibiotic resistance of bacterial strains. In the present study, graphitic carbon nitride nanosheets are prepared by controlled calcination of urea. Spectroscopic measurements show that the nanosheets consist of abundant carbonyl groups and exhibit apparent photocatalytic activity under UV photoirradiation towards the selective production of singlet oxygen. Therefore, the nanosheets can effectively damage the bacterial cell membranes and inhibit the growth of bacterial cells, such as Gram-negative *Escherichia coli*, as confirmed in photodynamic, fluorescence microscopy, and scanning electron microscopy measurements. The results from this research highlight the unique potential of carbon nitride derivatives as potent antimicrobial agents.

## 1. Introduction

Antibiotic resistance is a major threat to human health, causing a rift in the pursuit of effective treatment of bacteria-caused diseases [1,2,3,4]. Therefore, the development of high-performance antimicrobial materials has been attracting extensive interest. Among these, photoactive nanomaterials that can produce reactive oxygen species (ROS) under photoirradiation have been recognized as next-generation antimicrobial agents because ROS can attack bacterial cell membranes and DNA eventually leading to cell death [5,6,7,8]. There are various forms of ROS, such as singlet oxygen (^1^O_2_), superoxide radical ($O_{2}^{∙-}$), and hydroxy radical (OH^•−^). Among these, singlet oxygen, an electronically excited state of oxygen, has been attracting unique interest because of its high reactivity, electrical neutrality, and long lifetime. Singlet oxygen plays a key role in photodynamic actions against a wide range of pathogens through lipid peroxidation, protein oxidation, and nucleic acid damage [9]. Singlet oxygen can be produced photochemically via the type II mechanism, where a photosensitizer (e.g., methylene blue, rose Bengal, and porphyrins) absorbs photons of appropriate wavelength forming an excited triplet state before undergoing intersystem crossing and energy transfer to ground-state (triplet) oxygen [10]. For instance, back in 1990, Malik et al. [11] demonstrated the effective photosensitizing of porphyrin derivatives in the production of singlet oxygen and other ROS and their antimicrobial activities due to alterations in cell wall and membrane synthesis. Hill and coworkers employed oligo-*p*-phenylene ethynylene (OPE) as a UVA photosensitizer and generated singlet oxygen as a potent antimicrobial agent towards both Gram-negative and Gram-positive bacteria [12]. Singlet oxygen has also been produced by exploiting the photochemical properties of glutathione-capped Ag_31_ nanoclusters [13].

In a series of recent studies, graphitic carbon nitride (g-C_3_N_4_)-based materials have emerged as effective photocatalysts for the generation of singlet oxygen. For instance, Wang et al. [14] demonstrated that the triplet-exciton yield could be markedly improved by incorporating carbonyl (C=O) functional moieties into the g-C_3_N_4_ scaffold, in sharp contrast to pristine g-C_3_N_4_. Such a unique property could be exploited for select organic synthesis. This was ascribed to the carbonyl functionalization that boosted the spin-orbit coupling in g-C_3_N_4_ and hence facilitated the intersystem crossing of energy transfer [15]. Singlet oxygen can also be effectively produced via the type I pathway involving superoxide anions, as demonstrated in a recent study with S,K-codoped g-C_3_N_4_ in alkaline media [16], where molecular oxygen was reduced by photogenerated electrons to $O_{2}^{∙-}$, which was then oxidized by photogenerated holes to ^1^O_2_, leading to the effective degradation of targeted organic pollutants (e.g., bisphenol A).

In the present study, we demonstrate that the unique photocatalytic activity of g-C_3_N_4_ towards the selective production of singlet oxygen can be exploited for antimicrobial applications. Experimentally, g-C_3_N_4_ nanosheets were prepared by controlled calcination of urea, which featured abundant carbonyl functional groups and selectively produced singlet oxygen under UV photoirradiation, leading to effective inhibition of the growth of *Escherichia coli* (*E. coli*) bacterial cells, as confirmed in photodynamic, fluorescence microscopy, and scanning electron microscopy measurements.

## 2. Materials and Methods

### 2.1. Chemicals

Urea (Fisher Chemicals, Waltham, CA, USA), sodium chloride (NaCl, Fisher Chemicals), potassium chloride (KCl, Fisher Chemicals), sodium phosphate dibasic (NaH_2_PO_4_, Fisher Chemicals), potassium phosphate monobasic (Na_2_HPO_4_, Fisher Chemicals), Luria–Bertani (LB) broth (Fisher Chemicals), agar (Fisher Chemicals), 5,5′-dithio-bis-2-nitrobenzoic acid (DTNB, Fisher Chemicals), 5,5-dimethyl-1-pyrroline-N-oxide (DMPO, ACROS Organics, Geel, Belgium), glutathione (GSH, ARCOS Organics), tris-HCl (Sigma-Aldrich, St. Louis, MO, USA), methylene blue (MB, ARCOS Organics), and all solvents were obtained from typical commercial resources and utilized without further processing. Ultrapure water was obtained from a Barnstead Nanopure water system (resistivity 18.3 MΩ cm).

### 2.2. Sample Preparation

g-C_3_N_4_ nanosheets were prepared by following a procedure reported previously [17]. Briefly, 3 g of urea was placed onto a covered crucible and calcined in a tube furnace at 550 °C for 2 h under a constant nitrogen gas flow at a ramp rate of 5 °C min^−1^. 

### 2.3. Characterizations

Transmission electron microscopy (TEM) measurements were conducted on a Tecnai G2 (Waltham, Massachusetts, USA) operated at 200 kV, where the powder sample prepared above was dispersed in ultrapure water and drop-cast onto a holey carbon-coated copper grid. UV−vis measurements were performed on a PerkinElmer Lambda 35 UV−vis spectrometer (Waltham, Massachusetts, USA). X-ray photoelectron spectroscopy (XPS) measurements were conducted with a Thermo Fisher K-alpha (Waltham, Massachusetts, USA) instrument, where the binding energies were calibrated against the C 1s peak (284.4 eV). X-ray diffraction (XRD) patterns were collected using a Rigaku Ultima IV diffractometer (Wilmington, MA, USA) with the powder sample at a scan rate of 1° min^−1^. Raman measurements were conducted with a Horiba Jobin Yvon (Stow, MA, USA) LabRAM ARMIS automated scanning confocal Raman microscope under 532 excitation. For electron paramagnetic resonance (EPR) measurements, 63 μL of the sample prepared above (1 mg mL^−1^ in methanol) was mixed with 7 μL of DMPO (1 M), with a mixture of Nanopure H_2_O and DMPO as a control. One sample series was exposed to 365 nm light irradiation for 45 min, while another was in the dark. The solution was then loaded into a 1.5 mm OD capillary (Friedrich & Dimmock Borosilicate Capillary) for measurements. The tube was centered in the cavity resonator for data collection. Spectra were recorded at room temperature with a Bruker ElexSys E500 spectrometer (Billerica, MA, USA) operating at the X-band frequency (~9.8 GHz) using an ER 4122SHQE resonator at a microwave power of 20.03 mW, modulation amplitude of 1 G, modulation frequency of 100 kHz, and conversion time of 20.48 ms.

### 2.4. Ellman’s Assay

The Ellman method based on GSH oxidation was used to evaluate the oxidizing activity induced by the nanocomposite, as reported previously [18]. In brief, 5 mL solutions of the sample (160 μg mL^−1^) and equal amounts of GSH (1 mM) were prepared in phosphate buffer saline (PBS) 1× solution. The solutions were then mixed, shaken, and exposed to 365 nm UV light for 2 h. At select time intervals (e.g., 10 min), an aliquot of 450 μL was removed and mixed with 785 μL of a tris−HCl buffer (0.05 M, pH = 8.8) and 15 μL of the Ellman’s reagent DTNB (100 mM) and shaken for 1 min. The supernatant was collected post-centrifugation, and 200 μL of the supernatant was added to the 96-well plate, which was placed into a Molecular Devices SpectraMax Plus reader (San Jose, CA, USA). The wavelength of the enzyme marker was set at 410 nm to assess the GSH loss.

### 2.5. Photocatalytic Degradation of Methylene Blue

In this experiment, an MB solution was prepared by adding 6.5 mL of a 480 ppm MB stock to a PBS 1× solution to obtain a final volume of 100 mL (the PBS 1× solution was selected to maintain consistency across Ellman’s Assay and bactericidal studies). Then, 10 mL of the diluted MB dye was added to plastic scintillation vials along with 2.5 mg of the sample prepared above. The sample–dye solution was shaken at 1000 rpm while covered with tin foil in the dark for 1 h to reach an adsorption–desorption equilibrium before the solution was exposed to 365 nm photoirradiation with LEDs at an output of 1200 lumens. A series of aliquots (1 mL) was taken every 10 min, and the absorption spectra were acquired with a UV-vis spectrometer after centrifugation for 5 min to remove solid materials. The degradation activity was determined by normalizing the absorbance to that prior to photoirradiation. 

### 2.6. Photodynamic Studies

The procedure was detailed previously [18]. Experimentally, frozen glycerol stock *E. coli* (ATCC 25922) was streaked on an LB agar plate and incubated at 37 °C for 18 h. Then, one colony was selected for inoculation in 3 mL of LB broth and allowed to shake at 37 °C for 18 h on the following day. The sample was then centrifuged at 5000 rpm for 5 min and resuspended in a PBS solution (0.05 M, pH = 8.7) twice, and the optical density (OD) was set to 0.1 at 600 nm. Then, 1 mL of the bacterial suspension was added to 99 mL of PBS 1×. In the photodynamic antibacterial assessments, 100 μL of the previously mentioned suspension was transferred to a plastic scintillation vial, into which was added the carbon nitride sample prepared above (1 mg) with 9.9 mL of PBS at a total concentration of 0.1 mg mL^−1^. The scintillation vials containing the bacterial cells and samples were irradiated with a UVA light (365 nm) for various periods of time. From the diluted solution, 50 μL was plated and spread on LB agar plates using 5 sterile glass beads, which were incubated at 37 °C for 16 h. Then, the number of bacterial colonies forming units (CFUs) was counted by a plate reader (Acolyte Colony Counter, Fisher Scientific). Percent bacterial cell survival was determined by normalizing the CFUs to that prior to light exposure. All glassware that contacted bacterial suspensions was autoclaved to ensure sterilization and inhibit contamination.

### 2.7. Live/Dead Assay

The experimental parameters were selected according to the manual for the ThermoFisher LIVE/DEAD™ *Bac*Light™ Bacterial Viability Kit L13152. *E. coli* was inoculated and grown in LB media for 18 h, followed by two PBS 1× washes prior to experimentation. Then, the *E. coli* suspensions were adjusted to 0.12 OD at 670 nm. Bacterial suspensions were then added to PBS 1× containing 1 mg of sample, which was then exposed to 365 nm UV light for 45 min under constant shaking. Then, 100 μL of the sample along with 100 μL of STYO9/PI dye mixture was added to a 96 well plate for imaging on a Perkin Elmer Revvity Opera Phenix Plus (Waltham, MA, USA, RRID SCR_021114) with an excitation wavelength centered at about 485 nm (green) and another wavelength centered at about 530 nm (red) for each well of the entire plate. The corresponding bar graph was constructed with a MATLAB script to subtract fluorescence from the g-C_3_N_4_ sample and count cells according to green/red staining.

### 2.8. SEM Imaging of Bacterial Cells

The effect of g-C_3_N_4_ on *E. coli* morphology was examined by scanning electron microscopy (SEM) measurements at 10 kV in vacuum. In brief, *E. coli* was inoculated in 3 mL of LB broth and allowed to shake at 37 °C for 18 h. The bacterial suspension was then centrifuged at 5000 rpm for 5 min and resuspended in PBS (0.05 M, pH = 8.7) twice. Then, the bacterial sample was treated with 1 mg of carbon nitride under UV light irradiation for 45 min, and 5 μL was dropcast onto an aluminum disk for imaging with a Thermo Scientific Apreo SEM instrument using the in-lens secondary electron detector.

## 3. Results

**Figure 1:**
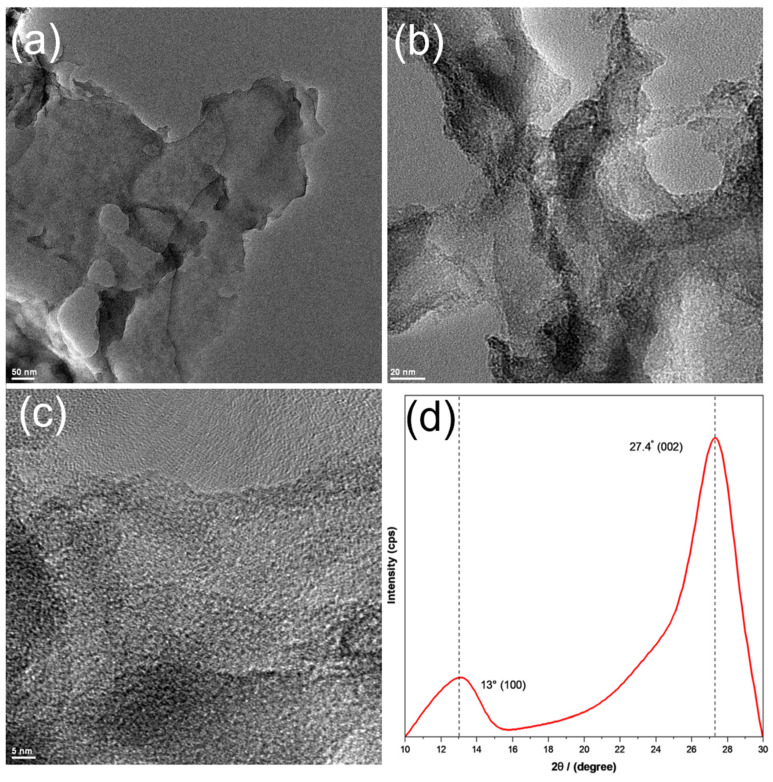
(**a**–**c**) Representative TEM images and (**d**) XRD patterns of g-C_3_N_4_. Scale bars are (**a**) 50 nm, (**b**) 25 nm, and (**c**) 5 nm.

The sample structure was first examined by TEM measurements. From the TEM images in Figure 1a,b, one can see that g-C_3_N_4_ displayed a porous sheet-like skeleton, consistent with the typical morphology of graphitic carbon nitride [19]. In high-resolution TEM measurements (Figure 1c), the sample can be found to possess a mostly amorphous structure, with only short-range lattice fringes (Figure S1), where two interplanar spacings of 0.41 and 0.71 nm can be resolved and ascribed to the (002) and (100) crystalline planes of graphitic carbon nitride (g-C_3_N_4_), respectively [19,20]. Consistent results were obtained in XRD measurements (Figure 1d), where the sample can be found to possess two broad diffraction peaks, a major one at 2θ = 27.4° and a minor one at 13.1°. The former can be ascribed to the (002) planes of g-C_3_N_4_, whereas the latter to the (100) diffraction because of the interlayer structural packing of g-C_3_N_4_ [19,20]. Additionally, based on the full width at half maximum of the (002) peak, the size of the crystalline domain was estimated by using the Scherrer equation to be ca. 3.1 nm, in good agreement with results from TEM measurements (Figure S1).

**Figure 2:**
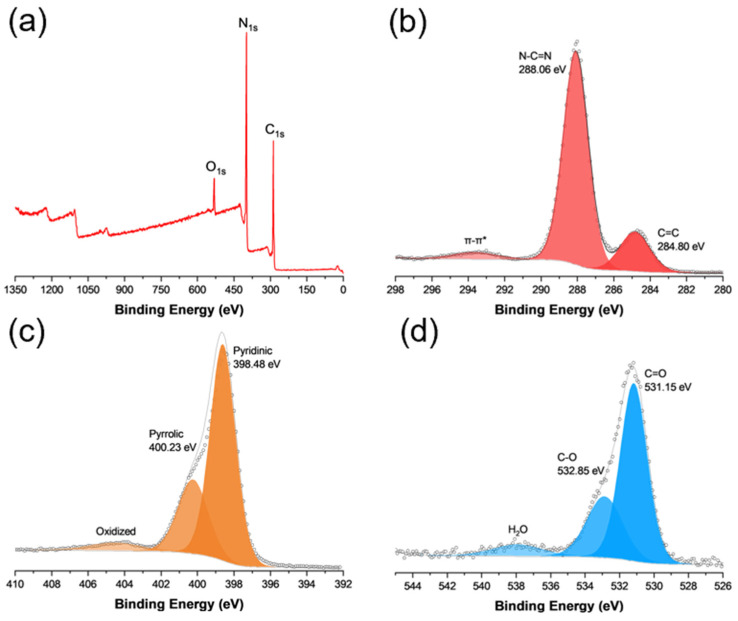
(**a**) Survey spectrum and high-resolution XPS spectra of the (**b**) C 1s, (**c**) N 1s, and (**d**) O 1s of g-C_3_N_4_. Solid curves are experimental data and shaded peaks are deconvolution fits.

The elemental composition and valence states of the g-C_3_N_4_ sample were then analyzed via XPS measurements. From the survey spectrum in Figure 2a, one can see that the C 1s, N 1s, and O 1s electrons can be readily resolved at ca. 284, 400, and 530 eV, respectively. From the C 1s spectra in Figure 2b, deconvolution yields two peaks, a major one at 288.06 eV for N-C=N and a minor one at 284.80 eV for sp^2^ C, in good agreement with results obtained in previous studies [21,22,23]. The corresponding N 1s spectrum is shown in Figure 2c, where three species can be deconvoluted at 398.50, 400.23, and 404.48 eV, due to pyridinic, pyrrolic, and oxidized nitrogen, respectively [24,25]. Notably, the atomic ratio of C in N-C=N to pyridinic N was estimated to be 1:1.24 for C_3_N_4_, close to that (1:1.33) of pristine C_3_N_4_ (Table S1) [26,27]. In addition, from the O 1s spectrum (Figure 2d), deconvolution yields two peaks at 531.15 and 532.85 eV. The former can be assigned to carbonyl C=O and the latter to C-O, suggesting (a) the formation of abundant carbonyl moieties within the C_3_N_4_ scaffold and (b) effective adsorption of oxygen species, both critical towards singlet oxygen production [14]. Good agreement was observed in Raman spectroscopic measurements (Figure S2) [16].

The optical properties of g-C_3_N_4_ were then examined via UV-vis diffuse reflectance spectroscopy (DRS) and photoluminescence measurements. Figure S3a depicts the UV-vis absorption spectra for the sample. One can see that C_3_N_4_ exhibited an absorption threshold of ca. 440 nm [28], corresponding to a band gap of ca. 2.82 eV (inset to Figure S2a), consistent with results reported previously for the g-h-heptazine phase of g-C_3_N_4_ [29,30]. In time-resolved photoluminescence measurements at the excitation of 400 nm (Figure S2b), the data can be fitted with a biexponential function, featuring an average emission lifetime (t) of ca. 1.07 ns.

**Figure 3:**
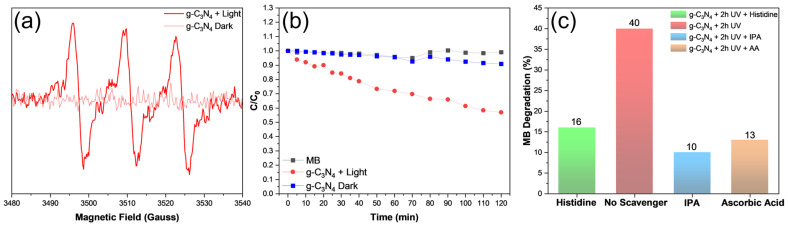
(**a**) EPR spectrum in the presence of g-C_3_N_4_ and DMPO after 45 min of 365 nm photoirradiation (bold curve) and in the dark (light curve). The stars identify the three peaks due to hyperfine splitting, indicative of singlet oxygen formation. (**b**) Photocatalytic degradation of methylene blue dye under UV photoirradiation in the presence and absence of g-C_3_N_4_. C_0_ is the initial dye concentration and C is the concentration at a specific time point (t). (**c**) Effects of scavengers on the degradation rate of methylene blue under UV photoirradiation for 2 h.

Notably, under UV photoirradiation, the obtained g-C_3_N_4_ efficiently catalyzed the selective production of singlet oxygen, as manifested in EPR measurements. From Figure 3a, a triplet hyperfine feature was observed between 3490 and 3540 G at the intensity ratio of ca. 1:1:1 and a g value of 2.0066, confirming the successful formation of singlet oxygen [31], in contrast to the featureless profile in the dark. The produced singlet oxygen can be exploited for the degradation of organic pollutants, as evidenced in Figure 3b. One can see that in comparison with the blank control or when the experiment was conducted in the dark, UV photoirradiation for 2 h led to ca. 40% removal of methylene blue, and the rate constant (k) was estimated to be 0.0045 min^−1^ by fitting the data with the first-order reaction kinetics, ln(C/C_0_) = −kt, where C_0_ is the initial methylene concentration and C is the concentration at a specific time point (t) (Figure S4) [6]. Notably, when histidine, isopropyl alcohol (IPA), and ascorbic acid (AA) were added into the methylene blue solution, the photocatalytic degradation efficiency decreased significantly to only 16% for histidine, 13% for AA, and 10% for IPA (Figure 3c). As these are the effective scavengers for ^1^O_2_ [32,33,34], the results further confirm the photocatalytic activity of the produced g-C_3_N_4_ in the selective production of singlet oxygen, most likely because of the abundant carbonyl moieties within the scaffold, as observed previously [14].

**Figure 4:**
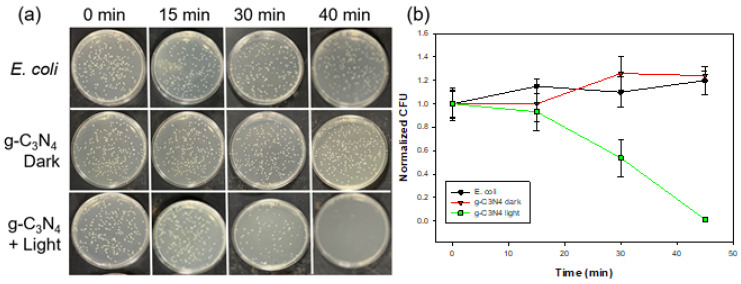
(**a**) Digital photographs of incubated LB agar plates from photodynamic bacterial experiments. (**b**) Photodynamic experiment of *E. coli* under UV light irradiation for 45 min. Colony forming units are normalized to the value at the 0 min timepoint.

Such unique properties can be exploited for antibacterial applications. In Figure 4a, one can see that in comparison with the blank control, the g-C_3_N_4_ nanosheets exhibited apparent inhibition of *E. coli* cell growth after ca. 15 min of UV photoirradiation and almost complete inhibition after 30 min. Such a performance is comparable to those observed earlier with metal-doped nanocomposites [35,36]. Note that *E. coli* growth was not impacted in the dark even in the presence of g-C_3_N_4_. Thus, the apparent diminishment in the bacterial CFUs in the presence of g-C_3_N_4_ under photoirradiation suggests the high photodynamic activity of the sample (Figure 4a), consistent with results from Ellman’s assay (Figure S5). 

**Figure 5:**
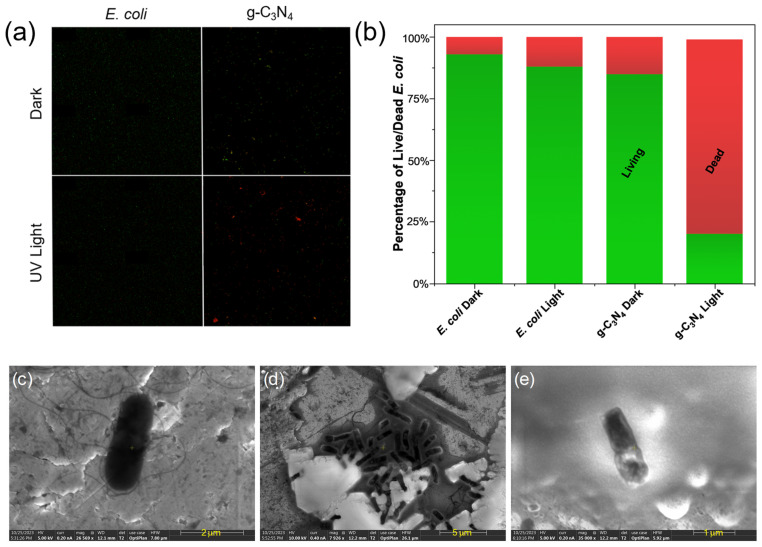
(**a**) Fluorescence microscopic image of *E. coli* and treated with samples before and after UV light irradiation for 45 min. Green fluorescence is indicative of living bacteria, whereas red fluorescence is indicative of dead bacteria. (**b**) Bar graph displaying CFU counts taken from the live/dead assay. SEM images of *E. coli* (**c**) before and (**d**,**e**) after photoirradiation with C_3_N_4_ for 45 min. Scale bars are (**c**) 2 μm, (**d**) 5 μm, and (**e**) 1 μm.

To validate the antibacterial activity of g-C_3_N_4_, a live/dead assay with SYTO9 and PI dyes was employed to detect living (green) and dead bacteria (red) with and without the g-C_3_N_4_ photocatalyst as well as with and without UV light. In the fluorescence microscopic images in Figure 5a,b, the *E. coli* control displayed a strong green fluorescence, corresponding to a normal living state of the bacterial cells. In contrast, almost all *E. coli* showed red fluorescence after UV light irradiation for 45 min in the presence of g-C_3_N_4_, suggesting effective cell wall damage of the bacteria cells, as red fluorescence arose from the binding of PI to the DNA of damaged bacterial cells [35,36].

To unravel the mechanistic insights, SEM measurements were then performed to examine the morphological changes in the bacterial cells in the absence and presence of g-C_3_N_4_ and with and without photoirradiation. A change in the bacterial morphology can be clearly seen in SEM measurements of *E. coli* (Figure 5c–e), where photo-irradiative treatment with g-C_3_N_4_ resulted in apparent damage to the bacterial cell membranes. Notably, in comparison with the *E. coli* cell grown in the absence of g-C_3_N_4_ (Figure S6), the bacterial cell can be seen to be virtually invariant in size and shape (Figure 5c), suggesting no effect of g-C_3_N_4_ alone on bacterial cell growth.

As mentioned earlier, the carbonyl-enriched g-C_3_N_4_ nanosheets facilitated the selective production of singlet oxygen under UV irradiation by energy transfer to atmospheric triplet oxygen (type II pathway) [14]; meanwhile, photogenerated electrons could also react with molecular oxygen to yield superoxide anions that then reacted with valence-band holes (h^+^) to produce singlet oxygen (type I pathway) [37]. As bacterial cells, such as *E. coli*, typically carry a negative surface charge, the accessibility of negatively charged superoxide radicals to cell membranes is most likely hindered by electrostatic repulsion [38]. Additionally, bacterial cells can develop resistance to superoxide radicals, but not singlet oxygen species, by upregulating defenses [32]. This suggests that singlet oxygen was likely the primary contributor to the antimicrobial action within the present experimental context.

## 4. Conclusions

In this study, graphitic carbon nitride nanosheets were prepared by controlled pyrolysis of urea featuring abundant carbonyl surface moieties. Under UV photoirradiation, the obtained g-C_3_N_4_ facilitated the efficient production of singlet oxygen. This led to apparent photocatalytic activity towards the degradation of organic dye and inhibition of the growth of *E. coli* cells. The results of this work highlight the unique potential of graphitic carbon nitride derivatives in the selective production of singlet oxygen and their application as effective bactericidal agents.

## Data Availability

Data are available upon request from the authors.

## Supplementary Materials

The following supporting information can be downloaded at https://www.mdpi.com/article/10.3390/ma17153787/s1. Figure S1. High-resolution TEM image of g-C_3_N_4_. Figure S2. Raman spectrum of g-C_3_N_4_. Figure S3. (a) UV-vis diffuse reflectance and (b) time-resolved photoluminescence spectra of C_3_N_4_ at the excitation of 400 nm. Inset in (b) shows lifetimes. Figure S4. Semi-log plot of methylene blue degradation by g-C_3_N_4_ under UV photoirradiation. Figure S5. Ellman’s Assay under (a) blue and (b) UV photoirradiation for 120 min. Control signifies the negative control glutathione, whereas g-C_3_N_4_ is depicted by the red bar. Figure S6. SEM image of an E. coli cell grown in the absence of g-C_3_N_4_. Table S1. Summary of XPS fitting results of g-C_3_N_4_.
