# Supplementary material for: Photocatalytic Generation of Singlet Oxygen by Graphitic Carbon Nitride for Antibacterial Applications
Source: Materials (Basel). 2024 Aug 1;17(15):3787. doi: 10.3390/ma17153787 (PMC11313655; doi:10.3390/ma17153787)
Supplement: Supplementary file 1 [file materials-17-03787-s001.zip › materials-3087374-supplementary.pdf]

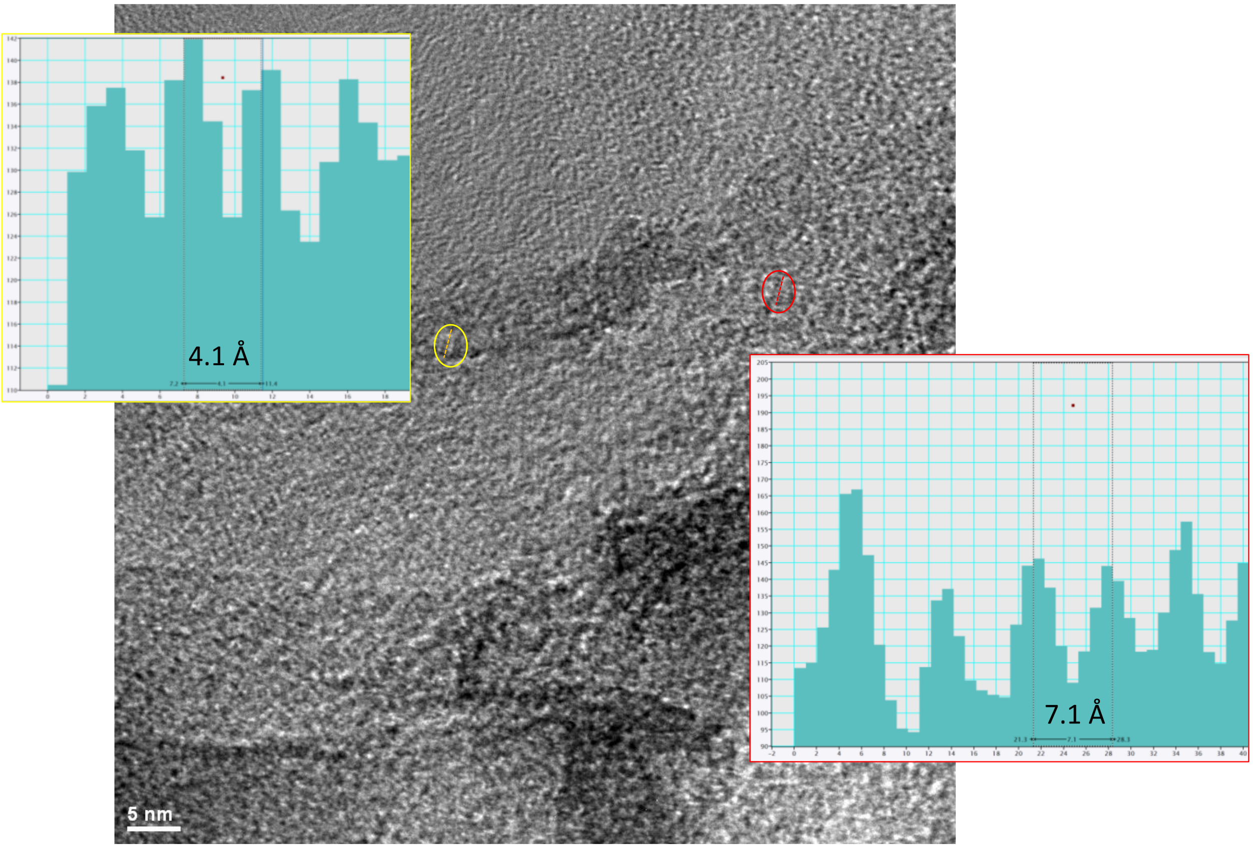

Figure S1. High-resolution TEM image of g-C_3N_4. Insets are the lattice fringe spacings of the yellow and red circle, respectively.

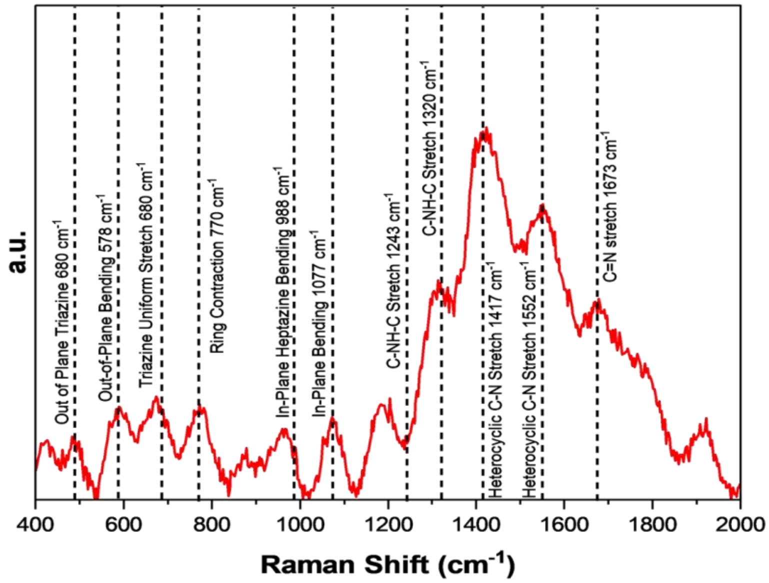

Figure S2. Raman spectrum of g-C_3N_4.

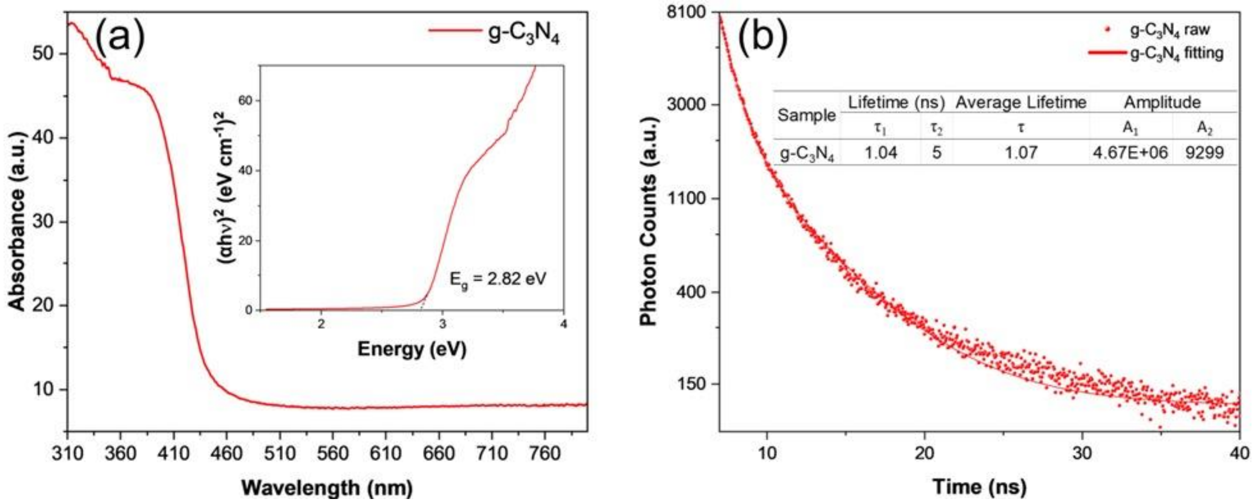

Figure S3. (a) UV-vis diffuse reflectance and (b) time-resolved photoluminescence spectra of C_3N_4 at the excitation of 400 nm. In panel (b), symbols are experimental data and line is biexponential fit, $y = a_1e^{-t/\tau 1} + a_2e^{-t/\tau 2}$. Inset to (b) shows the lifetimes.

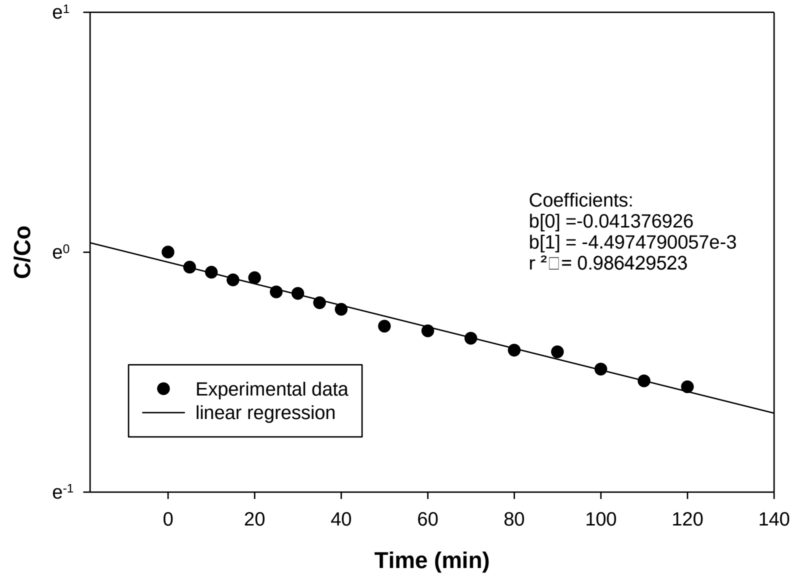

Figure S4. Semilog plot of the decay of methylene blue by g-C_3N_4 under UV photoirradiation. Symbols are experimental data from Figure 3b and line is linear regression.

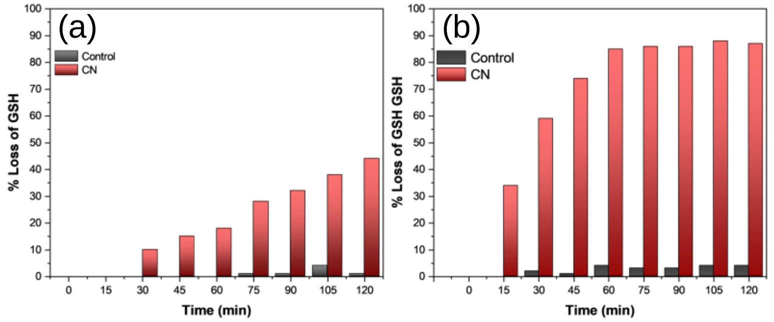

Figure S5. Ellman's Assay under (a) blue and (b) UV photoirradiation for 120 min. Control signifies the negative control glutathione whereas g-C_3N_4 is depicted by the red bar.

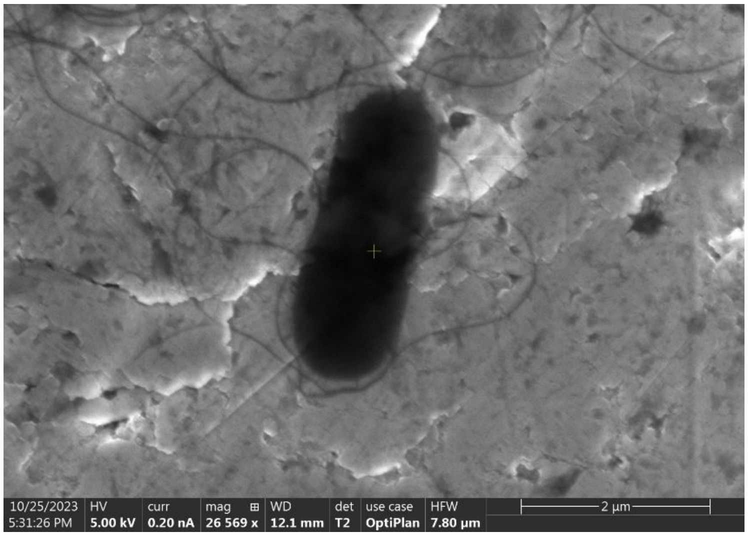

Figure S6. SEM image of *E. coli* grown in the absence of g-C_3N_4.

Table S1. Summary of XPS fitting results of g-C_3N_4.

g-C_3N_4	Species	BE (eV)	Atomic %	Weight %	Total Atomic %
C 1s	C=C	284.8	7.27	6.57	42.44
	N-C=N	288.06	33.33	30.13	
	π-π*	293.59	1.84	1.67	
N 1s	Pyridinic (C-N=C)	398.48	33.39	35.2	51.25
	Pyrrolic (N-C_3)	400.23	15.2	16.03	
	Oxidized N	404.48	2.66	2.81	
O 1s	C=O	531.15	4.02	4.85	6.3
	C-O	532.85	1.81	2.18	
	H_2O	537.89	0.47	0.57	
